# Supplementary material for: Are carbon emissions trading and green financial instruments synergistic? -Comprehensive quantitative research based on content analysis
Source: PLoS One. 2024 Mar 7;19(3):e0298601. doi: 10.1371/journal.pone.0298601 (PMC10919601; doi:10.1371/journal.pone.0298601)
Supplement: S1 Data — (ZIP) [file pone.0298601.s001.zip › Data/地级市Z_F工作报告.zip!/╣π╢1⁄2/║╙╘┤╩╨/└┤╫╘╣1⁄2╓┌║┼í╛┬φ┐╦╩2╛▌═°í┐_2012.docx]

各位代表：

现在，我代表市人民政府向大会作政府工作报告，请予审议，并请各位政协委员和其他列席人员提出意见。

过去五年工作回顾

　　市五届人大一次会议以来，本届市政府在省委、省政府和市委的正确领导下，在市人大及其常委会和市政协的监督支持下，以科学发展观统揽全局，全面贯彻落实党中央、国务院和省委、省政府一系列决策部署，按照市第五次党代会要求，积极践行“三反”理念，团结带领全市人民开拓创新、攻坚克难，较好地完成了本届政府确定的各项目标任务。

　　一、经济持续较快增长，综合实力明显增强。预计2011年，全市完成生产总值575.4亿元，比上年增长13.1%，五年年均增长13.8%；实现全社会工业总产值1197亿元、增加值275.3亿元，分别增长21.6%、18%，比2006年翻了一番多，年均分别增长23.1%、19.8%；地方财政一般预算收入31.4亿元，增长32.8%，比2006年翻了一番多，年均增长20.2%；五年累计完成固定资产投资1084亿元，比上五年增长1.32倍；实际利用外资1.79亿美元，增长6%；外贸出口总额19.4亿美元，增长13%，比2006年增长2.5倍，年均增长28.3%；全社会消费品零售总额188亿元，增长15.1%，比2006年翻了一番多，年均增长16.7%；城镇居民人均可支配收入14534元，增长10.3%，年均增长8.8%；农村居民人均纯收入6660元，增长18%，年均增长10.3%。民营经济快速发展，民营经济增加值占全市生产总值比重近六成。实施富县强镇战略，县域综合实力持续增强。

　　二、“四新”产业快速发展，产业结构明显优化。产业转型升级加快，初步建立了以“四新”产业为主导的现代产业体系，三大产业结构由2006年的14.8∶48.4∶36.8优化为13.0∶53.1∶ 33.9。 园区建设实现新突破。2011年，“一区六园”完成工业总产值635亿元，五年增长2.18倍，成为全市经济发展重要增长极。成功创建5个省级产业转移园，成为全省最多的市。其中中山（河源）产业转移园实现工业总产值250亿元，五年增长3.3倍，两次竞得省扶持资金共10亿元，连续多年被省评为优秀等次，被列为“省手机产业集群升级示范区”、“省首批外贸转型升级专业型示范基地”、“省市共建河源太阳能光伏产业基地”。2011年手机产量达4439万台、产值155亿元。落户园区的广东汉能薄膜太阳能电池项目试产，中兴通讯、旗滨光伏玻璃等项目动工建设。 现代农业取得新成效。2011年，农林牧渔业实现总产值121亿元，五年增长24.7%。新增省级以上农业标准化示范区18个、农业龙头企业134家，创建了“广东省供港鲜活农产品外贸转型升级专业型示范基地”，成功举办了2届市农业博览会。“菜篮子”和“米袋子”工程建设顺利推进。东江上游特色水果产业带初具规模。 生态旅游呈现新亮点。“五大”旅游品牌知名度明显提升，2011年接待游客1336万人次，旅游总收入59亿元，五年分别增长1.67倍和1.64倍。改造了万绿湖等一批主要景区景点，开发了赵佗故城、林寨古村等一批新景区，引进了东江·DD庄园、东江源温泉度假村、康泉18国际生态旅游城等一批大型旅游产业项目，创建国家4A级旅游景区2家；新建五星级酒店1家、四星级3家，在建五星级3家。 现代服务业实现新发展。2011年，第三产业实现增加值195.3亿元，五年年均增长11.7%。市区商业中心动工建设。房地产、金融保险、商贸流通、中介信息等取得长足发展。 科技创新能力得到新提升。建成国家通讯终端产品质量监督检验中心等一批公共技术服务平台。

　　三、基础设施不断完善，城乡面貌明显改观。人居环境指数在中国社科院《2011年城市蓝皮书》排位中居全国第4位。 城乡规划不断完善。在全省率先开展“三规合一”规划编制工作试点，开展了新一轮《河源市城市总体规划》、《河源市土地利用总体规划》修编，编制了《河源市城乡总体规划》、《河源市公园城市专项规划》、《河源市万绿水城专项规划》和《河源市东江、新丰江两江四岸城市总体设计》等一批重大规划。市广电中心投入使用并获全市首个“鲁班奖”。 交通建设成效显著。五年新增公路里程1326公里，公路密度由86.4公里/百平方公里提高到93.2公里/百平方公里。汕湛、东环、大广、汕昆、粤湘高速公路前期工作扎实推进。市区“六路一桥”、大环市路龙川到紫金段建成通车，市区永和路、纬十一路、纬十二路、大同路等“断头路”贯通，学前坝、群丰和白岭头等片区“水浸街”问题有效解决。 电力建设步伐加快。河源电厂一期工程建成运营。建成首座500千伏变电站，25个输变电工程建成投产，新增35千伏及以上变电容量383万千伏安、35千伏及以上送电线路1209公里，圆满完成电网建设和农网改造任务。 城管水平大幅提升。城管体制进一步理顺，成功创建国家卫生城市，城市面貌焕然一新。改造和新建一批供水管网，城市公交系统实现改造升级。五年改造和新建农贸市场17个，正在改造和新建8个。初步建成梧桐山公园，改造提升了笔架山和龟峰公园。市区建成区绿化覆盖率由40.4%提高到44.2%。 新农村建设稳步推进。五年建设新农村示范点413个，新建硬底化村道3600公里，通行政村道路全面实现硬底化。宜居村镇“六个一”工程扎实推进。7宗15项城乡水利防灾减灾工程基本完工，完成166座中小型水库除险加固，在全省率先开展中小河流治理工作。农田水利等重点工程顺利推进。解决了20.5万农村人口饮水困难和76万人饮水安全问题。

　　四、生态环保力度加大，环境优势明显提升。被评为“广东省园林城市”，创建4个省级林业生态县，通过了国家环保模范城市省级预评估，连平县被评为全国生态建设示范县。 监管力度不断加大。五年淘汰落后钢铁产能362万吨、落后水泥产能101万吨，连续五年完成节能减排任务。依法关闭非法矿点500多个。 环保设施逐步完善。五年建成9座污水处理厂（日处理污水能力22.5万吨）和1座生活垃圾卫生填埋场，城市生活污水集中处理率、工业废水排放达标率、生活垃圾无害化处理率分别由58.78%、93.56%、0提高到89%、100%、99.66%。 生态建设明显加强。积极创建林业生态市，在全省率先完成林业体制改革、率先实行森林防火问责制和全面实施封山育林，基本完成集体林权制度改革任务，森林覆盖率达 71.7%。

　　五、文化教育实现突破，社会事业明显进步。高中阶段教育毛入学率达86.34%，五年提高近30个百分点，提前一年实现“普高”目标。成功举办中国首届客家文化节和世界客属第23届恳亲大会。 创建教育强市扎实推进。坚持优先发展教育，五年新建河源理工学校、东江中学，扩建河源职业技术学院、市高级技工学校等66所学校，创建国家级示范性普通高中4所、国家级重点中等职业学校4所、省义务教育规范化学校128所，3个镇成功创建省教育强镇。 文化建设实现重大进展。大力弘扬“两地”文化，完成第三次全国文物普查，新增国家非物质遗产名录2项，保护和修缮了一批文化遗址和革命旧址，建成市恐龙博物馆等一批文化设施。市被评为“省文明城市”，龙川县被认定为“千年古县”和“中央苏区县”，连平县城被评为“全国文明县城”，源城区被省评为“南粤锦绣工程”文化先进区。 其他社会事业全面进步。深入开展医药卫生体制改革，建成市人民医院新院、市妇幼保健院新院、市中医院新院、市职业病和慢性病防治院等一批骨干医院，基本完成乡镇卫生院标准化改造，有效缓解了群众看病难问题。连续两次成功创建全国双拥模范城。全面完成人口计生目标，稳定低生育水平，源城区被评为“国优”，4个县被评为“省优”。实现亚运会、全运会金牌零的突破。完成全国经济、农业和人口普查等统计调查任务。被评为“全国人民防空先进单位”。救灾减灾、社区建设、基层政权、社会组织、人事编制、民族宗教、外事侨务、驻外机构、妇女儿童、科协、对台、气象、打私、供销、档案、关工委、老龄、残联、“双拥”人民武装等各项事业取得新成绩。

　　六、“双十”工程全面完成，民生福祉明显改善。五年来各级财政的民生投入达361.4亿元，完成本届政府承诺办好的“十项惠民工程”和每年办好的“十件实事”。 扶贫“双到”成效显著。累计投入扶贫“双到”资金超过17亿元，省认定贫困村集体经济年收入全面达到3万元，完成贫困村和省属水库移民农房改造任务，85%在册贫困户达到脱贫标准，省扶贫考核获“优秀”等次。理顺移民管理体制，落实移民后扶政策，改善移民生产生活环境，完成广东（东源）水库移民“双转移”示范基地首期建设。全面落实“千村脱困”政策，1126个贫困村每年共获分红款2600多万元。 社会保障体系不断完善。扎实做好就业再就业工作，五年培训农村劳动力13.6万人，新增转移就业32.5万人，城镇登记失业率控制在2.1%。参加企业职工养老、失业、医疗、工伤、生育保险人数五年分别增长53%、47%、117%、50%、60%。新农合参合率达98.9%，五年提高37.6个百分点。新农保试点取得实效，城镇居民养老保险正式启动，低保覆盖面逐步提高。住房保障工作稳步推进，五年有效解决2.7万人住房困难。健全价格调节基金和低收入群众临时价格补贴机制，缓解了低收入群众生活困难。 社会管理卓有成效。三级综治信访维稳平台全面建成使用，网上信访工作有效推进，信访维稳工作得到加强。深入推进公安机关社会管理“六项创新”，重拳打击涉黑涉恶、涉枪涉爆、涉毒涉赌、“两抢一盗”、拐卖妇儿以及网络犯罪等各类犯罪行为，圆满完成“平安亚运、和谐世客”等重大活动安保任务，2010年公众安全感和公安工作群众满意度均居全省第二位。越级上访批数和人次五年分别下降36.2%和14.6%。安全生产、产品质量和食品药品监管、地质灾害防治、应急管理及处置等工作得到增强。

　　七、科学发展能力提高，政府建设明显加强。切实加强队伍建设，加大问责督办力度，推动各项工作扎实开展。认真执行市人大及其常委会各项决议并向其报告工作，坚持向市政协通报情况，代表建议、政协提案办复满意和基本满意率超过99%。完成新一轮政府机构改革和第四轮行政审批制度改革，行政审批项目纳入电子监察系统，实行实时全程监控。认真落实党风廉政建设责任制和领导干部廉洁自律各项规定，党风廉政建设多年在全省考核中名列前茅。

　　各位代表，回顾过去五年发展历程，奋斗充满艰辛，成绩令人鼓舞，经验弥足珍贵。我们深切体会到： 必须始终坚持把加快发展作为首要任务，才能从根本上改变河源相对落后的状况，争取实现率先发展； 必须始终坚持把生态发展作为重要原则，不断提升生态水平，推动经济与生态协调持续发展，才能在剧烈竞争中凸现科学发展新优势； 必须始终坚持把招商选资作为主要抓手，锲而不舍抓招商、上项目，才能夯实产业基础和增强发展后劲，保持良好发展态势； 必须始终坚持把优化政务环境作为重要保障，切实转变机关作风，不断提高服务水平，才能增强广大投资者的发展信心，吸引国内外发展要素向本地集聚； 必须始终坚持把改善民生作为根本目的，切实解决好群众切身利益问题，让全市人民共享发展成果，政府工作才能得到群众的拥护和支持； 必须始终坚持把新时期河源人精神作为动力源泉，充分调动各方积极性和创造性，才能在率先崛起的征程中保持不竭动力。

　　各位代表！过去五年我市取得的成绩来之不易。这是省委、省政府和市委正确领导的结果，是全市人民团结拼搏、共同奋斗的结果，也离不开深圳、中山等兄弟市和海内外各界人士的关心支持。在此，我代表市人民政府，向全市广大干部群众，向驻河源部队、消防官兵和各民主党派、人民团体、社会各界人士致以崇高的敬意！向长期关心支持我市改革发展的港澳同胞、台湾同胞、海外侨胞表示衷心的感谢！

　　在看到成绩的同时，我们也清醒地看到存在的困难和问题： 一是加快发展任务仍然艰巨，如经济总量太小，财政收支矛盾突出，支撑加快发展的工业和县域经济发展不够快； 二是工业园区承接能力不强，招商引资力度不足，氛围不浓，引进的项目数量不多、质量不高，实际利用外资欠理想，园区生产力和财源培植速度偏慢； 三是又好又快发展的要求越来越高，发展的难度也越来越大，发展瓶颈问题更加突出，如土地、资金、人才、技术等要素制约越来越明显，生态保护和节能减排的压力不断增大； 四是保障和改善民生任务繁重，如卫生、社保、社会救助等水平偏低，社会事业建设欠账较多，基本公共服务均等化水平不高； 五是社会管理存在薄弱环节，土地管理、矿产管理、环境管理等一些问题和矛盾亟待解决； 六是干部作风有待进一步改进，有些干部发展意识不强，不思进取，安于现状，尤其缺乏攻坚克难、创新进取的精神；有些干部主动服务意识淡薄，办事效率不高，部门办事难现象仍然存在，行政效能和服务水平有待提升；有些干部作风不够扎实，责任心和执行力不强，工作不推不动，甚至推而不动，抓落实的工作机制有待加强，特别是乡镇干部中存在严重的“走读”现象，等等。对这些问题，我们要高度重视，采取措施，认真加以解决。

今后五年奋斗目标和工作要求

　　今后五年，是我市加快科学发展、建设幸福河源的重要时期。当前，我市经济社会发展面临良好机遇： 一是有总体向好的外部环境。全球经济结构加快调整，科技创新和产业转型正孕育突破；国家确定了“稳中求进”工作基调，实施“稳增长、控物价、调结构、惠民生、抓改革、促和谐”工作方针，继续实施积极的财政政策和稳健的货币政策，为我市加快发展营造了良好的外部环境。 二是有加快发展的坚强支撑。省委、省政府深入实施“双转移”、扶贫开发“双到”、基本公共服务均等化等政策，将进一步加大对山区发展的扶持。 三是有乘势而上的良好基础。近年来推进了一批重大基础设施、引进了一批重大产业项目，将产生积极的带动作用。同时，也面临严峻挑战： 一是国际金融危机还没有结束，世界经济增长在短中期内可能呈低迷状态，下行风险明显增加，不利于我市外资经济发展。 二是国内发展进入增速减缓和转型加速时期，劳动力、土地、能源资源等要素价格刚性上涨，生态环境保护任务艰巨，我市加快发展的难度加大。 三是周边地区你追我赶，发展迅猛，我市“奋起直追、争先进位”的压力更加凸显。但总体来说，机遇大于挑战。我们一定要增强忧患意识、使命意识和责任意识，克服安于现状、畏难不前的思想，转变敷衍应付、不推不动的作风，振奋精神、攻坚克难、开拓进取，始终牢牢把握工作主动权，努力推动经济社会加快发展。

　　基于以上考虑，今后五年政府工作的指导思想是：坚持以中国特色社会主义理论体系为指导，全面贯彻落实中央、国务院和省委、省政府一系列决策部署，按照市第六次党代会要求，以科学发展为统揽，以加快发展为第一要务，以新型工业化为主攻方向，以增进民生福祉为执政根本，全力实施生态经济、宜居城乡、善治河源、文化河源“四大战略”，加快经济、生态、文化“三大崛起”，努力走出一条具有山区特色的生态文明发展、社会和谐善治、人民共建共享的幸福河源之路。

　　围绕上述指导思想，今后五年政府工作的奋斗目标是：全面完成“十二五”规划目标，到2016年全市生产总值超1100亿元、全社会工业总产值超2200亿元、地方财政一般预算收入超70亿元，年均分别增长15%、15%、18%以上；城镇居民人均可支配收入年均增长9%以上，农村居民人均纯收入年均增长13%以上；努力创建教育强市、全国双拥模范城（县）、国家环保模范城市、国家园林城市；经济发展跃上新台阶、城乡建设焕发新面貌、文化事业呈现新繁荣、社会管理开创新局面、幸福河源谱写新篇章。

　　围绕奋斗目标，今后五年政府工作要正确处理好“六大关系”： 一是坚持好快并重。坚定不移地把加快经济发展摆在更加突出的重要位置，同时正确处理好与快的关系，做到好字当头、好中求快，不断提高发展质量。 二是坚持新旧兼顾。加快转变发展方式，进一步培育发展战略性新兴产业、现代农业和现代服务业；同时正确处理新与旧的关系，在大力引进新项目的基础上，积极帮扶现有企业增资扩产、做大做强，做到新旧兼顾、协调发展。 三是坚持“星月同辉”。牢牢扭住项目建设不放松，以项目建设大突破来推动经济社会大发展；同时正确处理内与外、大与小的关系，既注重引进外资项目又重视推动民营经济发展，既注重引进大项目又重视引进中小项目，做到以外促内、以大带小、“星月同辉”。 四是坚持绿富双赢。全面加强环境保护和生态建设，进一步巩固和提升生态优势；同时正确处理绿与富的关系，既要绿起来，又要富起来，做到生态发展、绿富双赢。 五是坚持城乡共荣。拓展城市空间，提升城市功能，进一步提升城区的首位度；同时正确处理城与乡的关系，充分发挥城对乡的辐射带动作用，做到以城促乡、城乡共荣。 六是坚持严活互补。切实加强土地、矿产、森林、水等资源管理；同时正确处理严与活的关系，在规范管理的基础上，科学有序开发优势资源，做到严而不死、活而不乱。全力实施好“六大工程”： 一是实施生态经济发展工程，进一步提高产业发展水平； 二是实施重大设施建设工程，进一步改善发展条件； 三是实施生态环境保护工程，进一步构建山区发展优势； 四是实施文化河源建设工程，进一步提升文化软实力； 五是实施公共服务均等化工程，进一步保障和改善民生； 六是实施社会管理创新工程，进一步推进善治河源建设。
